# Lung tumor segmentation methods: Impact on the uncertainty of radiomics features for non-small cell lung cancer

**DOI:** 10.1371/journal.pone.0205003

**Published:** 2018-10-04

**Authors:** Constance A. Owens, Christine B. Peterson, Chad Tang, Eugene J. Koay, Wen Yu, Dennis S. Mackin, Jing Li, Mohammad R. Salehpour, David T. Fuentes, Laurence E. Court, Jinzhong Yang

**Affiliations:** 1 Department of Radiation Physics, The University of Texas MD Anderson Cancer Center, Houston, Texas, United States of America; 2 The University of Texas Graduate School of Biomedical Sciences at Houston, Houston, Texas, United States of America; 3 Department of Biostatistics, The University of Texas MD Anderson Cancer Center, Houston, Texas, United States of America; 4 Department of Radiation Oncology, The University of Texas MD Anderson Cancer Center, Houston, Texas, United States of America; 5 Department of Radiation Oncology, Shanghai Chest Hospital, Shanghai Jiao Tong University, Shanghai, China; 6 Department of Imaging Physics, The University of Texas MD Anderson Cancer Center, Houston, Texas, United States of America; University of Pennsylvania Perelman School of Medicine, UNITED STATES

## Abstract

**Purpose:**

To evaluate the uncertainty of radiomics features from contrast-enhanced breath-hold helical CT scans of non-small cell lung cancer for both manual and semi-automatic segmentation due to intra-observer, inter-observer, and inter-software reliability.

**Methods:**

Three radiation oncologists manually delineated lung tumors twice from 10 CT scans using two software tools (3D-Slicer and MIM Maestro). Additionally, three observers without formal clinical training were instructed to use two semi-automatic segmentation tools, Lesion Sizing Toolkit (LSTK) and GrowCut, to delineate the same tumor volumes. The accuracy of the semi-automatic contours was assessed by comparison with physician manual contours using Dice similarity coefficients and Hausdorff distances. Eighty-three radiomics features were calculated for each delineated tumor contour. Informative features were identified based on their dynamic range and correlation to other features. Feature reliability was then evaluated using intra-class correlation coefficients (ICC). Feature range was used to evaluate the uncertainty of the segmentation methods.

**Results:**

From the initial set of 83 features, 40 radiomics features were found to be informative, and these 40 features were used in the subsequent analyses. For both intra-observer and inter-observer reliability, LSTK had higher reliability than GrowCut and the two manual segmentation tools. All observers achieved consistently high ICC values when using LSTK, but the ICC value varied greatly for each observer when using GrowCut and the manual segmentation tools. For inter-software reliability, features were not reproducible across the software tools for either manual or semi-automatic segmentation methods. Additionally, no feature category was found to be more reproducible than another feature category. Feature ranges of LSTK contours were smaller than those of manual contours for all features.

**Conclusion:**

Radiomics features extracted from LSTK contours were highly reliable across and among observers. With semi-automatic segmentation tools, observers without formal clinical training were comparable to physicians in evaluating tumor segmentation.

## Introduction

Precision medicine aims to customize cancer treatment for an individual patient by considering combined knowledge (i.e., conventional factors such as age and sex, genetics, proteins, and others) [1,2]. Precision medicine seeks to completely characterize the tumor to determine optimal treatment based on patient-specific characteristics. In recent years, studies have shown that radiomics features have the potential to significantly improve our ability to stratify patients according to likely treatment response beyond conventional prognostic factors, thereby leading to truly personalized cancer care [3–7].

The generic workflow of radiomics studies includes four steps: (1) image acquisition, (2) tumor delineation, (3) feature extraction, and (4) feature analysis [8,9]. The tumor delineation can be drawn manually or generated with a semi-automatic tool. Once the tumor delineation has been established, radiomics features are extracted from the tumor-defined region within the image. Thousands of radiomics features can be calculated for one tumor, and each feature characterizes the tumor in a different way. For example, roundness is a radiomics feature that characterizes the tumor shape and can be used to predict how the tumor may spread out to nearby locations. Lastly, features are evaluated to see whether they correlate with prognostic or predictive factors. Features that are shown to be predictive are then used to build outcome models that help predict how a patient will respond to a treatment. For different diseases, different radiomics features can be selected for outcome modeling to predict likely treatment response.

Before radiomics features can be clinically useful, it is necessary to investigate and understand the uncertainties of radiomics features. One major source of uncertainty comes from the tumor delineation. To manually delineate the tumor precisely, in general, is difficult. Tumors often lay adjacent to other organs that share similar characteristics with the tumor, making it difficult to distinguish the true tumor boundary. Additionally, medical images are far from perfect, as they have limited resolution (limiting our ability to see very small objects) and can contain artifacts (features in an image that do not represent a real aspect of the imaged object). Physicians may interpret the tumor differently, depending on their training and experience [10]. In addition, the different software tools that physicians use to draw the tumor contours may also affect the results, depending on user familiarity with the tool. Because radiomics features are calculated from the delineated tumor, uncertainty in tumor delineation could propagate to the radiomics features.

Recent advances in computer-aided automatic and semi-automatic segmentation approaches have been shown to reduce the burden in manual delineation and lessen the inconsistency in tumor delineation [11,12]. To date, a small number of studies have been performed to relate this reduced uncertainty in tumor delineation to the quality and reproducibility of radiomics features [13–17].

In this current study, we examined three specific factors that can influence the uncertainty of radiomics features for both manual and semi-automatic segmentation methods: (1) intra-observer, (2) inter-observer, and (3) inter-software. Manual contours were generated by three independent physicians using MIM Maestro^TM^ (MIM Software Inc., Cleveland, Ohio, USA) and 3D-Slicer [18]. Semi-automatic contours were generated by three trained observers using the GrowCut algorithm from 3D-Slicer [11] and the Lesion Sizing Toolkit (LSTK) [19]. While the segmentation accuracy of LSTK has been evaluated [19,20], to our knowledge the reliability of radiomics features extracted from LSTK-generated contours has not been studied. Additionally, we evaluated whether manual software tools and semi-automatic software tools can be used interchangeably for generating contours for feature extraction. The purpose of this study can be summarized into two main objectives. The first objective was to identify a reliable segmentation tool that produces lung tumor segmentations that yield reliable and robust radiomics features for the same observer, across multiple observers, and across multiple software tools. The second objective was to identify a group of reliable radiomics features for non-small cell lung cancer (NSCLC) primary tumors.

## Materials and methods

### Patient data and CT image acquisition

**Fig 1:**
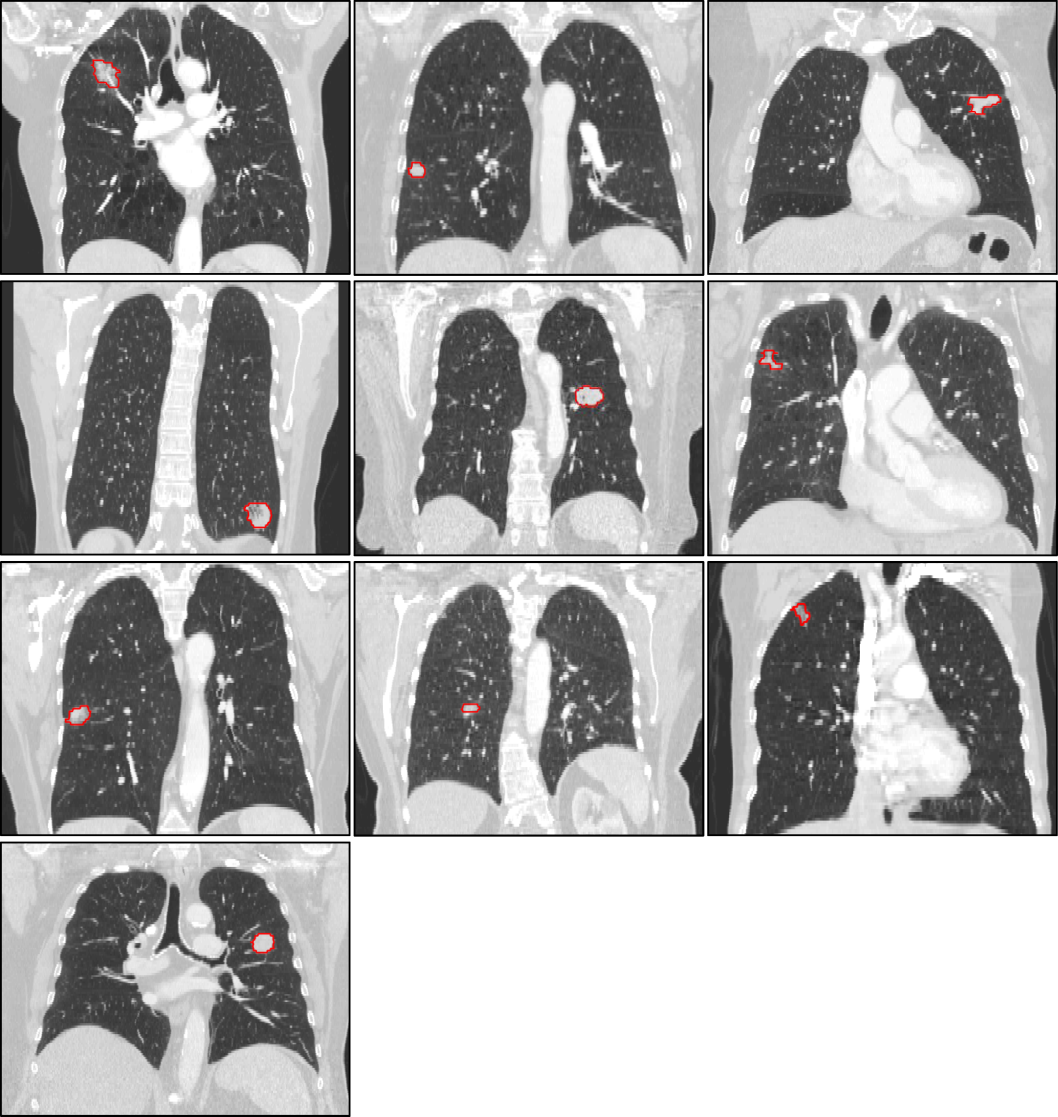
Tumor presentations and locations. A central slice of each tumor in the coronal view is displayed to show the variety in tumor locations, shapes and appearances of the patients used in this study. A single physician contour is displayed (red) to identify the tumor in each patient scan.

For this study, we retrospectively obtained patient data for 10 patients with histologically verified NSCLC. The Institutional Review Board (IRB) at the University of Texas MD Anderson Cancer Centers approved the present retrospective study, and the requirement for informed consent was waived. The lung tumors included in this study had volumes ranging from 1.15 cm^3^ to 10.53 cm^3^. For each patient, breath-hold helical computed tomography (CT) scans were acquired with intravenous contrast. The CT scans were acquired on General Electric Healthcare CT scanners with a peak tube voltage of 120 kVp and tube currents ranging from 320 mAs to 570 mAs. Each scan was reconstructed with a slice thickness of 2.5 mm and pixel spacing between 0.635 mm and 0.977 mm. Fig 1 shows a coronal slice of each tumor to display the variety of tumor presentations and locations of this patient cohort.

### Manual segmentation

**Fig 2:**
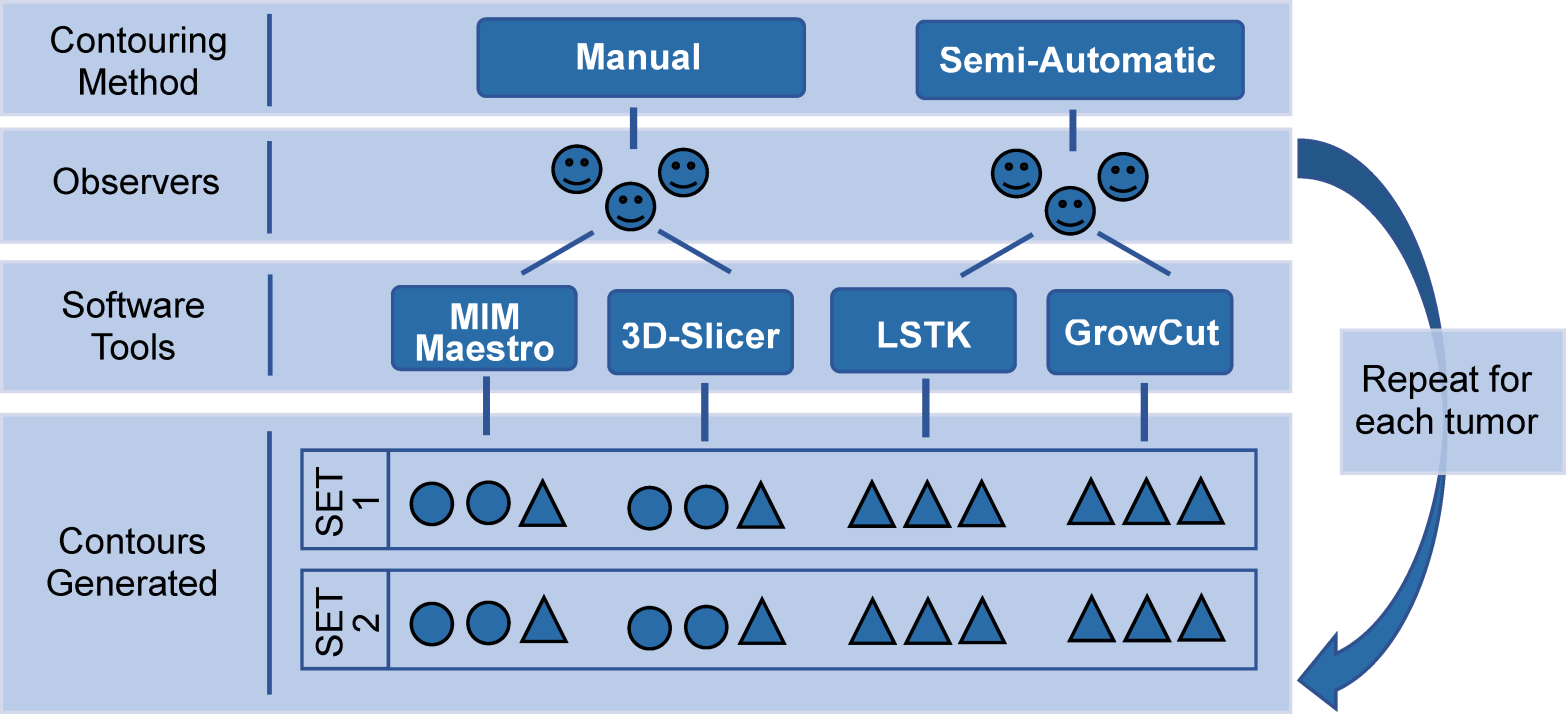
Schematic of the collection of manual and semi-automatic contours. Each circle and triangle represent a single tumor contour. The time interval between contour set 1 and contour set 2 was 1 year for the contours represented by circles and 1 month for the contours represented by triangles.

Manual segmentations were performed by three radiation oncologists using two different software tools: MIM Maestro^TM^ (MIM Software Inc., Cleveland, Ohio) and 3D-Slicer (a free open-source software platform) [18]. Each physician manually segmented each of the 10 tumors using both manual software tools, following the RTOG 1106 contouring guideline [21,22]. This guideline recommends contouring the primary tumor volume on CT images using a standard lung window/level for distinguishing lung borders and using a mediastinal window/level for distinguishing borders adjacent to the mediastinum. This process was repeated twice at two different times, yielding two sets of contours (Fig 2). The time intervals between the two sets of contours for each physician were approximately 1 year for the first two physicians and 1 month for the third physician. In total, 120 manual tumor contours were generated (2 software tools × 3 observers × 2 contours × 10 tumors). For both manual software tools, tumors were contoured using a paintbrush tool (thresholding in 3D-Slicer) in a slice-by-slice fashion in the transverse plane. Physicians could observe and edit the tumor in the coronal and sagittal planes as well, when desired.

### Semi-automatic tumor segmentation

Semi-automatic segmentations were generated using two different software tools: LSTK (a level-set algorithm available from an open-source toolkit) and GrowCut (a region growing algorithm implemented in 3D-Slicer). For the semi-automatic segmentations, three observers without formal clinical training were instructed to use the two semi-automatic tools to generate tumor segmentations. Verbal step-by-step instructions were given to each observer on using each software tool. After that, observers practiced using each software tool on three lung tumors (outside the study). The entire process took less than 15 minutes, with instruction lasting 5 minutes and practice lasting less than 10 minutes. Once observers felt comfortable with the software tool, the segmentations for this study were collected. The contouring process that was used for the manual contours was repeated for the semi-automatic contours for the same 10 tumors (Fig 2). The time interval between the two sets was 1 to 2 months for each observer to lessen memory effects. Other studies showed that 3 weeks between contouring runs are enough to mitigate the effects of memory [23].

**Fig 3:**
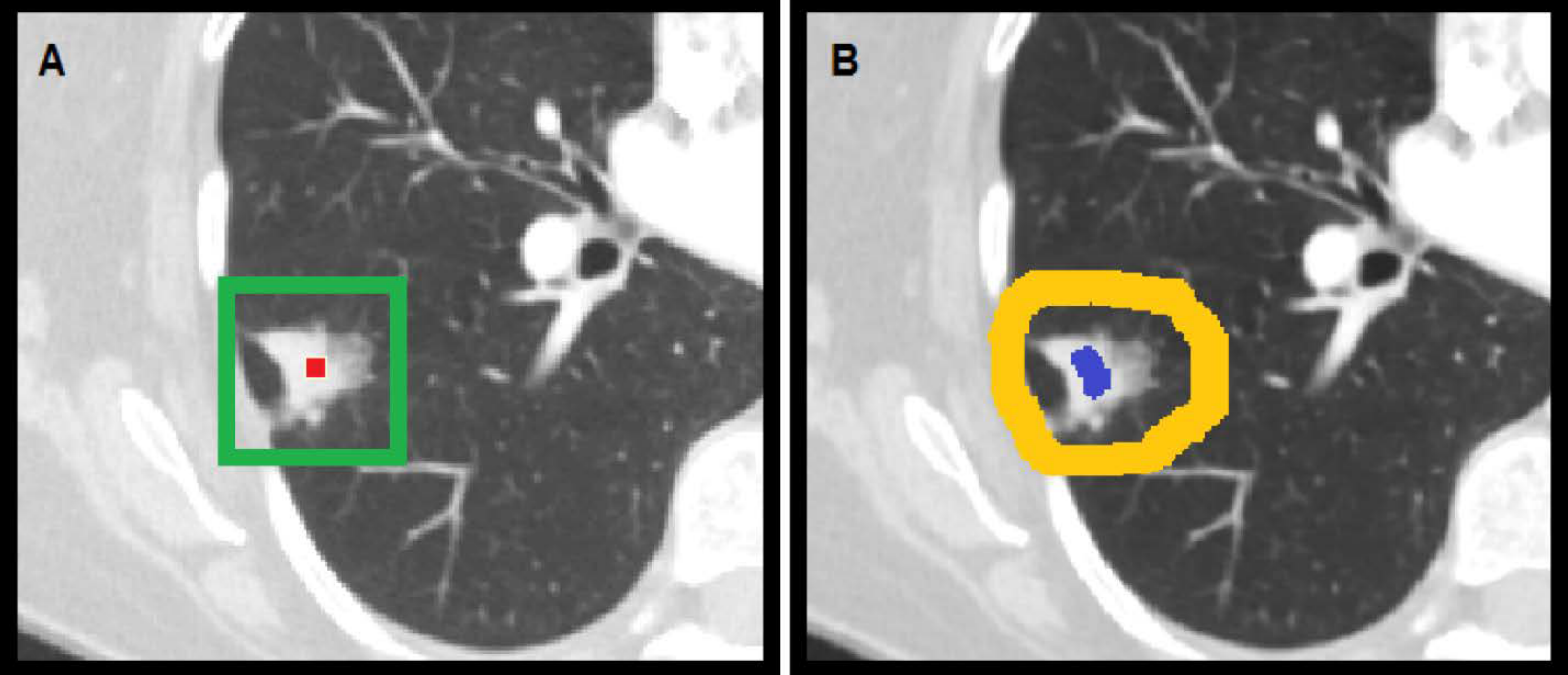
User inputs for initializing semi-automatic segmentation tools. (A) LSTK requires the user to select a seed within the tumor (red) to initiate the segmentation algorithm. Defining the maximum tumor radius generates a 3D bounding box (green) centered about the seed, within which the segmentation result will be confined. (B) GrowCut requires the user to label foreground (blue) and background (yellow) pixels to initiate the segmentation algorithm. Once labels were established, the GrowCut algorithm was followed by manual editing of the GrowCut-generated contours. Note that only the transverse view is shown here. Observers also labeled foreground and background pixels in the coronal and sagittal planes for each tumor case.

For GrowCut, observers labeled foreground and background pixels with two clicks (Fig 3) in each view, totaling in at least six clicks per tumor case. If the tumor was attached to the chest wall or mediastinum, additional clicks at appropriate location are needed to help the algorithm differentiate the tumor from the chest wall or mediastinum. Once labels were established, the GrowCut algorithm was followed by manual editing of the GrowCut-generated contours. The editing process took up to 2 minutes for some tumor cases.

For LSTK, the only interaction was to pick a seed which is a user-selected voxel within the tumor (Fig 3). Defining the maximum tumor radius was optional; however, defining an appropriate maximum tumor radius might save computation time in running LSTK. The LSTK algorithm has several preset parameters that can affect the segmentation result. We used the initial physician manual contours to guide us in selecting these parameters. Detailed discussions regarding the algorithms of GrowCut and LSTK can be found in other publications [19,20].

### Validating tumor segmentation accuracy

We validated the accuracy of each semi-automatic segmentation. A group-consensus contour was generated as the ground truth where the group-consensus contour is taken to be the intersecting tumor volume shared by a majority of experts [23–25]. In this study, the group-consensus contour consisted of the tumor region where at least four of the initial six manual physician contours overlapped. To assess the accuracy of each tumor segmentation, the Dice similarity coefficient (DSC) and Hausdorff distance (HD) were calculated between the group-consensus contour and each individual semi-automatic contour. The DSC quantifies the spatial overlap between two contours, while the HD quantifies the longest contour distance between the boundaries of two contours. While the DSC can detect incorrectly labeled voxels, the HD metric is better at detecting deviations (sharp spikes or tiny holes) that significantly alter the contour shape but do not substantially alter the volume.

### Feature extraction

Features were calculated for all 240 tumor segmentations (120 manual + 120 semi-automatic). For this study, feature extraction was performed using the open-source Imaging Biomarker Explorer (IBEX) software [26]. A total of 83 features were calculated. We stratified the features into three main categories: geometric shape (SHP), intensity histogram (HIS), and texture (TXT). Co-occurrence matrix features (a subcategory of texture features) were calculated in four directions (0, 45, 90, and 135 degrees), and the final value was taken to be an average of these four directions to avoid directional bias [27]. A common pre-processing step used to refine contours before feature extraction is to remove voxels with intensity values for normal lung tissue, bone, or air that might be inside the tumor contour. Since the purpose of this study is to investigate the segmentation uncertainty on radiomics features, we omitted this step to adhere to the original segmentation. We also did not correct for pixel size [28] or perform smoothing [29] to avoid introducing other uncertainties to this study.

### Feature reduction

One common approach for narrowing the feature set is to apply a combination of different methods in a sequential manner [9,14,15,30,31] to remove features that are non-informative or redundant. In the current study, we applied two steps to reduce the initial feature set of 83 features to 40 informative and non-redundant features. The first step was to remove features that did not vary across different patients. For a feature to be informative, it must exhibit a range of values across different patients [9,14]. In other words, it must have a wide dynamic range to differentiate patients. Because multiple contours were generated for each patient, the average feature value was calculated for each patient. Before calculating the normalized dynamic range (NDR) for each feature, the average values for each feature were rescaled (across the patients) to have a mean of 0 and a standard deviation of 1 using z-score normalization, so that features with values of different scales could be compared. The NDR for each feature, *NDR*_*f*_, was calculated as:

$${NDR}_{f}=max(\hat{f_{avg}})-min(\hat{f_{avg}})$$

where $max(\hat{f_{avg}})$ is the maximum normalized average feature value across all patients and $min(\hat{f_{avg}})$ is the minimum normalized average feature value across all patients. Once the NDR is calculated for each feature, a cutoff value is chosen as a means to remove the least informative features. In general, the cutoff value is chosen arbitrarily and may be set to a higher or lower value [9,15]. For the second step, highly correlated features were removed. It is well known that many features are highly correlated [9]. To deal with this issue, we computed a correlation matrix to identify highly correlated features. In this step, Spearman correlation coefficients were computed to evaluate the correlation between all features.

### Feature reliability analysis

In this study, we examined three specific factors that can influence feature reliability: intra-observer, inter-observer, and inter-software (Table 1). Intra-observer agreement is a reliability measure of repeatability, while inter-observer and inter-software agreement are reliability measures of reproducibility [32]. To assess feature reliability, intraclass correlation coefficients (ICCs) were calculated for each feature. There are ten different forms of the ICC [33] and selecting the appropriate form depends on the experimental setup. To assess intra-observer reliability, we used a one-way random-effects model where the tumor cases are a random effect. To assess inter-observer and inter-software reliability, we used a two-way mixed-effects model where the tumor cases are a random effect and the observers (for inter-observer) and the software tools (for inter-software) are a fixed effect. The specific ICC form used to assess each reliability relationship is shown in Table 1. The ICC values, which can range from values of -1 to values of 1, were stratified into four different classifications. ICC values less than 0.4, between 0.4 and 0.6, between 0.6 and 0.75, and greater than 0.75 represented the ICC bounds for the classifications of poor, fair, good, and excellent reliability [23].

**Table 1:** ICC formulas used to assess feature reliability.

Reliability Factor	ICC Description^a^	ICC Equation^a^^,^ ^b^	Explanation of Reliability Factor Being Examined
**Intra-observer**	One-way random-effects model, single measure, absolute-agreement	$\frac{{MS}_{R}-{MS}_{W}}{{MS}_{R}+(k+1){MS}_{W}}$	To determine whether features can be extracted reliably from tumor contours generated by a single physician/observer using a single software tool at *multiple timepoints*
**Inter-observer**	Two-way mixed-effects model, single measure, absolute-agreement	$\frac{{MS}_{R}-{MS}_{E}}{{MS}_{R}+(k-1){MS}_{E}+\frac{k}{n}({MS}_{C}-{MS}_{E})}$	To determine whether features can be extracted reliably from tumor contours generated by *multiple physicians/observers* using a single software tool
**Inter-software**	Two-way mixed-effects model, single measure, absolute-agreement	$\frac{{MS}_{R}-{MS}_{E}}{{MS}_{R}+(k-1){MS}_{E}+\frac{k}{n}({MS}_{C}-{MS}_{E})}$	To determine whether features can be extracted reliably from tumor contours generated by a single physician/observer using *multiple software tools*

MS_R_ = mean square for rows; MS_W_ = mean square for residual sources of variance; MS_E_ = mean square error; MS_C_ = mean square for columns; n = number of tumors; k = number of physicians/observers.

^a^ The information and equations in these columns were taken from McGraw and Wong [33].

^b^ Each row represents a different tumor case and each column represents a different measurement (for intra-observer), different judge (for inter-observer), or different software tool (for inter-software).

#### Correlation between ICC and CCC

Concordance correlation coefficients (CCCs) were also calculated because other feature reliability studies have used the CCC metric in their analysis [14,29,34,35]. Spearman rank correlation coefficients and pairwise scatterplots were computed between the ICC and CCC estimates for each reliability relationship.

#### Identifying reliable feature categories

For this part of the analysis, we wanted to determine whether a specific feature category (shape, histogram, texture) was significantly more reproducible than another feature category. For this determination, Wilcoxon rank sum test (aka Mann-Whitney test) values were computed between each feature category combination (e.g., shape versus histogram) for each ICC relationship.

### Feature range analysis

For segmentations from each software tool, we calculated the feature range (inter-patient variability) across observers for each radiomics feature. First, we normalized each feature using z-score normalization. This allowed us to more easily compare and plot features on different scales. Each normalized feature, $\hat{f_{i}}$, was calculated as:

$$\hat{f_{i}}=\frac{f_{p,i}-{\bar{f}}_{p}}{\sigma_{p,f}}$$

where *f*_*p*,*i*_ is the feature for contour *i* from patient *p*, ${\bar{f}}_{p}$ is the mean value for feature *f* for all contours from patient *p*, and *σ*_*p*,*f*_ is the standard deviation for feature *f* for all contours from patient *p*. Then we recorded the minimum and maximum normalized feature values for each segmentation method to assess the feature range of each segmentation method.

## Results

### Validating tumor segmentation accuracy

**Fig 4:**
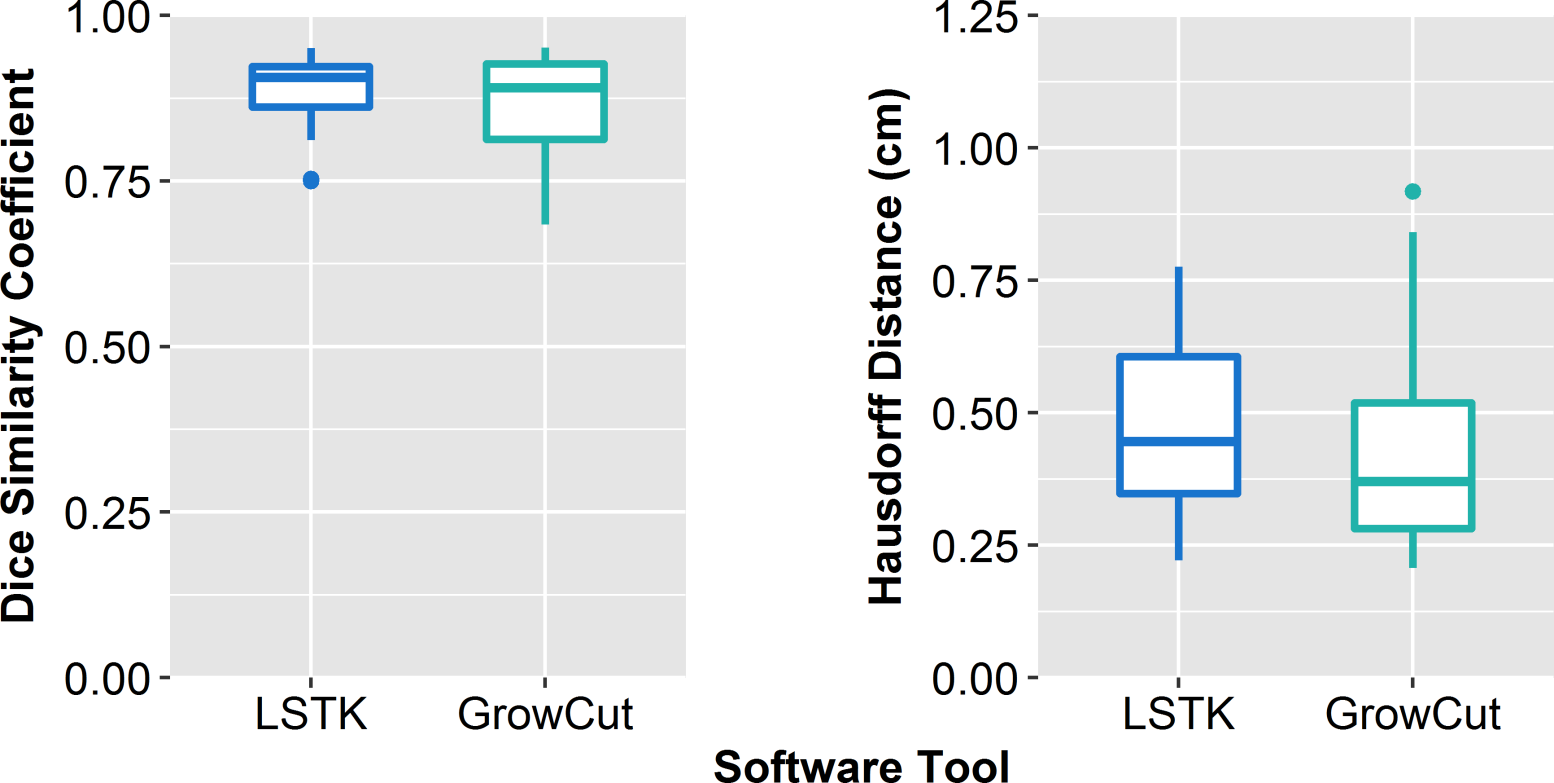
Validating segmentation accuracy of semi-automatic contours. Box plot of the Dice similarity coefficients and Hausdorff distances by software tool displays the segmentation accuracy for each software tool.

For the semi-automatic tools, the mean DSCs were 0.88 ± 0.06 and 0.88 ± 0.08 for LSTK and GrowCut, respectively (Fig 4). For the semi-automatic tools, the mean HD values were 0.48 ± 0.17 cm and 0.43 ± 0.20 cm for LSTK and GrowCut, respectively. The DSC and HD results show that trained observers can achieve comparable contours with these semi-automatic tools to the group-consensus physician contour, and hence these semi-automatically generated contours can be used for feature extraction.

### Feature reduction

**Fig 5:**
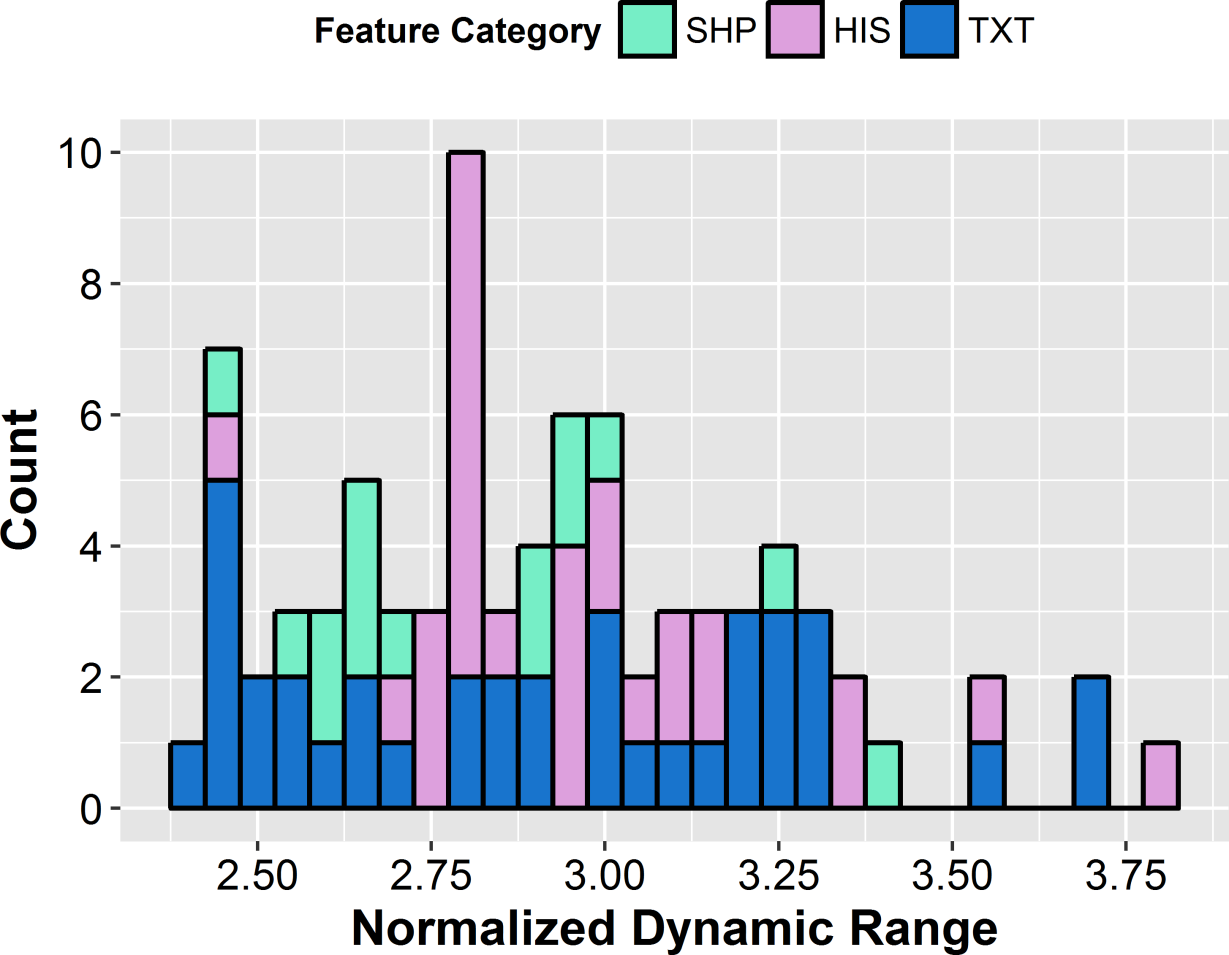
Histogram distribution of the normalized dynamic range for all 83 radiomics features. The histogram distribution shows the number of features within a range of NDR values where each bin has a width of 0.05.

**Fig 6:**
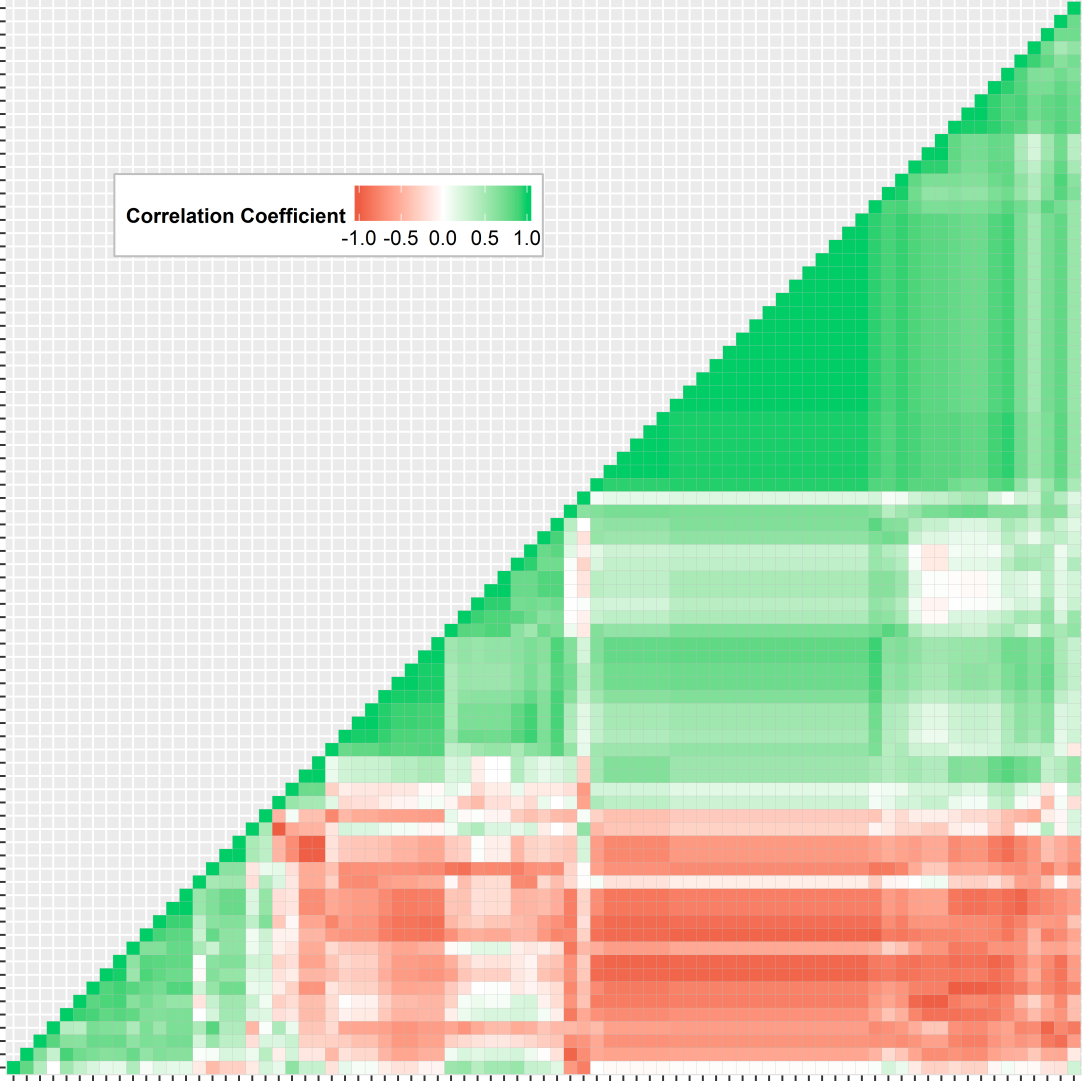
Spearman correlation coefficient heat map including all initial 83 features. Spearman correlation coefficients were computed for 83 radiomics features. Green, white, and red denote positive, random, and negative correlations, respectively. A large number of features were highly correlated.

**Fig 7:**
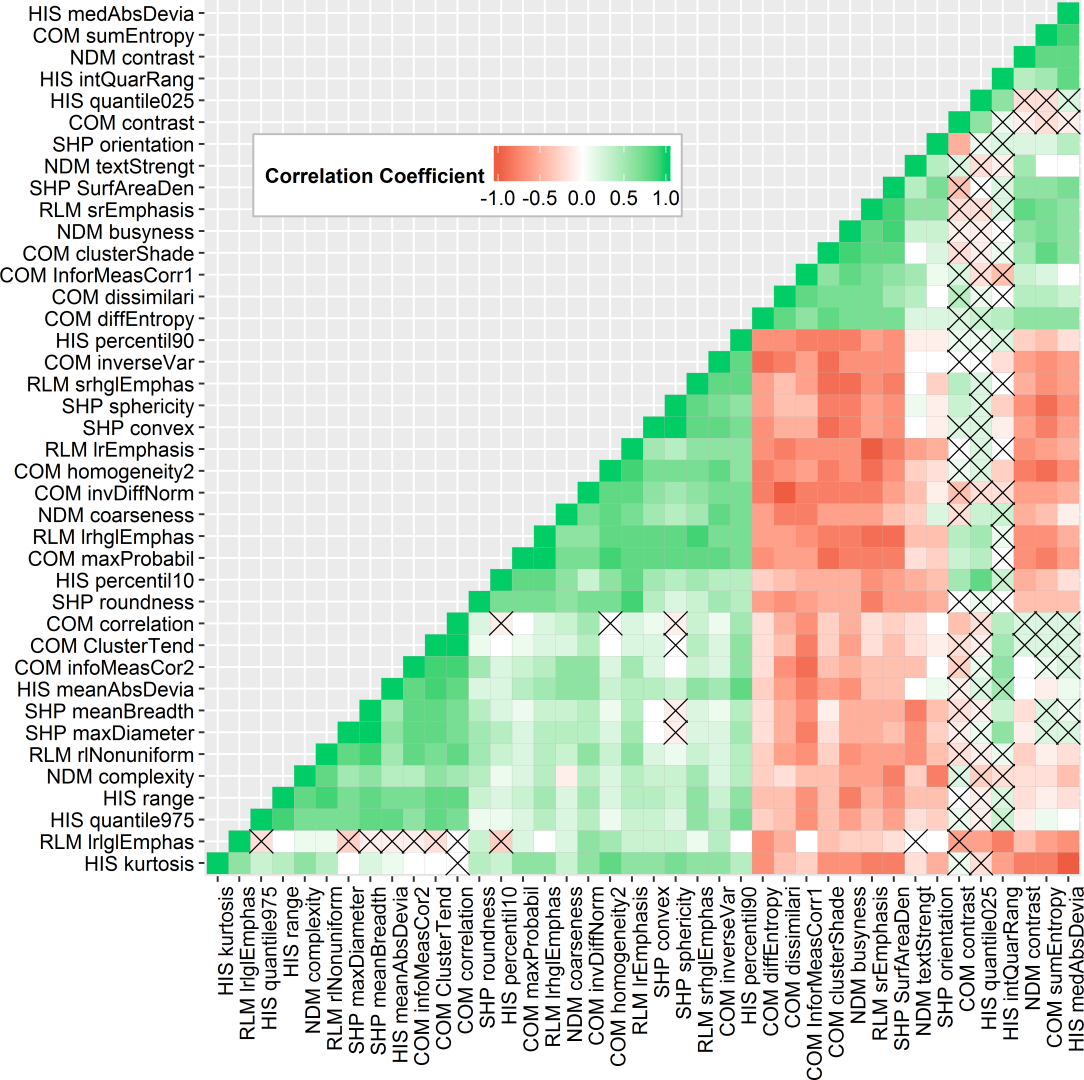
Spearman correlation coefficient heat map including 40 non-redundant features. Feature pairs with Spearman correlation coefficients less than 0.95. Spearman correlation coefficients larger than 0.95 were regarded as highly redundant and were eliminated from the initial feature set, reducing the feature set to 40 non-redundant features. Green, white, and red denote positive, random, and negative correlations, respectively. Correlation coefficients marked with an x are insignificant coefficients.

To identify non-informative features, the NDR was calculated for each feature. A histogram showing the number of features within a range of NDR values is shown in Fig 5. All features had an NDR value greater than 2.4 and hence all features were considered to exhibit large enough inter-patient variability to remain in the feature set. To evaluate the correlation between all features, pair-wise Spearman correlation coefficients were computed (Fig 6). Pair-wise correlation coefficients with an absolute value larger than 0.95 were regarded as very redundant [15]. For correlated features, the feature with the largest mean absolute correlation was removed, reducing the feature set to 40 non-redundant features (Fig 7).

### Feature reliability analysis

#### Correlation between ICC and CCC

For all reliability relationships, the results for the Spearman rank correlation coefficients between the CCC and ICC values showed a strong and statistically significant positive correlation (ρ>0.965, p<0.0001), indicating that feature reliability ranking was nearly the same for these two reliability metrics. For the pairwise scatterplots, all reliability relationships could be modeled with a strong positive linear regression fit line (R^2^>0.982, p<0.0001). These results indicate that the ICC and CCC metrics will yield similar results for analysis.

#### Feature repeatability: Intra-observer

**Fig 8:**
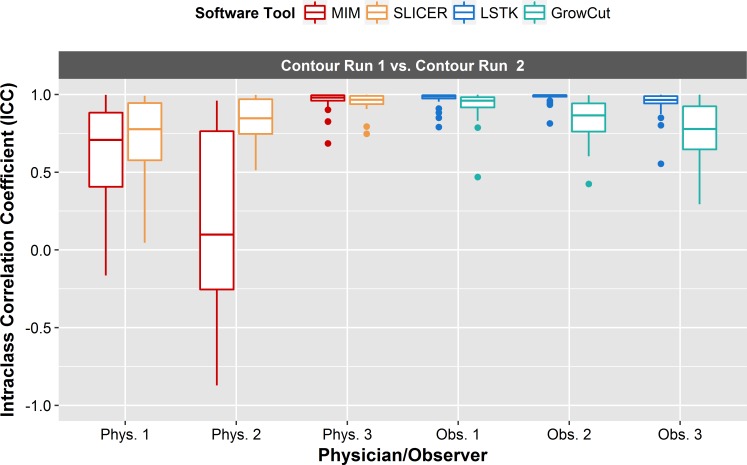
Intra-observer reliability. Box plot of ICCs for each intra-observer relationship. ICC values were computed between contour run 1 and contour run 2 for each feature. Each physician/observer and software tool combination is plotted along the *x*-axis. Intra-observer reliability was observer-dependent. All observers achieved excellent feature reliability with LSTK.

For intra-observer reliability, we wanted to evaluate whether features could be extracted reliably from tumor contours generated by a single observer using a single software tool at multiple time points. For each feature, ICC values were calculated between the features generated from the first and second contour runs for each user and software tool combination. The results showed that intra-observer reliability was highly observer dependent (Fig 8, Table 2). For the manual tools, the average ICC values were much lower for physicians 1 and 2 (MIM: 0.63, 0.17, 3DS: 0.72, 0.83) than the average values for physician 3 (MIM: 0.96, 3DS: 0.96). This is likely due to the fact that the time between the contour runs for physicians 1 and 2 was 1 year, whereas for physician 3 the elapsed time between contour runs was 1 month. For the semi-automatic tools, all observers achieved higher average ICC values with the software tool LSTK (0.97, 0.98, 0.85) than with GrowCut (0.94, 0.85, 0.75). This shows that LSTK can be used to minimize the effect from intra-observer variability compared with GrowCut, as was shown with observer 3 whose average ICC value improved substantially from 0.75 (for GrowCut) to 0.95 (for LSTK). LSTK requires less user interaction than GrowCut, which typically requires manually editing after the segmentation, thus leading to more consistent feature values and achieving better consistency.

**Table 2:** ICCs and confidence intervals.

**Intra-Observer**					
>**Segmentation Method**	>**Contour Run**	>**Software Tool**	>**Phys./ Obs.**	>**Mean ICC**^**a**^	>**Mean Confidence Interval**^**a**^
Manual	1 vs. 2	MIM	Phys. 1	0.63	(0.17, 0.88)
Manual	1 vs. 2	MIM	Phys. 2	0.17	(-0.29, 0.61)
Manual	1 vs. 2	MIM	Phys. 3	0.96	(0.86, 0.99)
Manual	1 vs. 2	SLICER	Phys. 1	0.72	(0.30, 0.92)
Manual	1 vs. 2	SLICER	Phys. 2	0.83	(0.52, 0.96)
Manual	1 vs. 2	SLICER	Phys. 3	0.96	(0.84, 0.99)
Auto	1 vs. 2	GrowCut	Obs. 1	0.94	(0.79, 0.98)
Auto	1 vs. 2	GrowCut	Obs. 2	0.85	(0.55, 0.96)
Auto	1 vs. 2	GrowCut	Obs. 3	0.75	(0.36, 0.93)
Auto	1 vs. 2	LSTK	Obs. 1	0.97	(0.90, 0.99)
Auto	1 vs. 2	LSTK	Obs. 2	0.98	(0.94, 1.00)
Auto	1 vs. 2	LSTK	Obs. 3	0.95	(0.82, 0.99)
**Inter-Observer**					
**Segmentation Method**	**Contour Run**	**Software Tool**	**Phys./ Obs.**	**Mean ICC**^**a**^	**Mean Confidence Interval**^**a**^
Manual	1	MIM	ALL	0.58	(0.30, 0.84)
Manual	1	SLICER	ALL	0.67	(0.39, 0.89)
Auto	1	GrowCut	ALL	0.70	(0.45, 0.89)
Auto	1	LSTK	ALL	0.98	(0.94, 0.99)
Manual	2	MIM	ALL	0.53	(0.23, 0.81)
Manual	2	SLICER	ALL	0.79	(0.55, 0.94)
Auto	2	GrowCut	ALL	0.85	(0.66, 0.96)
Auto	2	LSTK	ALL	0.96	(0.89, 0.99)
**Inter-Software**					
>**Segmentation Method**	>**Contour Run**	>**Software Tool**	>**Phys./ Obs.**	>**Mean ICC**^**a**^	>**Mean Confidence Interval**^**a**^
Manual	1	MIM-SLICER	Phys. 1	0.72	(0.32, 0.92)
Manual	1	MIM-SLICER	Phys. 2	0.43	(-0.04, 0.74)
Manual	1	MIM-SLICER	Phys. 3	0.75	(0.25, 0.92)
Manual	1	GrowCut-LSTK	Obs. 1	0.74	(0.31, 0.93)
Manual	1	GrowCut-LSTK	Obs. 2	0.76	(0.37, 0.93)
Manual	1	GrowCut-LSTK	Obs. 3	0.56	(0.15, 0.83)
Auto	2	MIM-SLICER	Phys. 1	0.52	(0.04, 0.83)
Auto	2	MIM-SLICER	Phys. 2	0.61	(0.20, 0.87)
Auto	2	MIM-SLICER	Phys. 3	0.72	(0.26, 0.92)
Auto	2	GrowCut-LSTK	Obs. 1	0.74	(0.34, 0.93)
Auto	2	GrowCut-LSTK	Obs. 2	0.78	(0.35, 0.94)
Auto	2	GrowCut-LSTK	Obs. 3	0.72	(0.24, 0.91)

^a^ Reported values are averages of their respective estimate for all 40 features.

#### Feature reproducibility: Inter-observer

**Fig 9:**
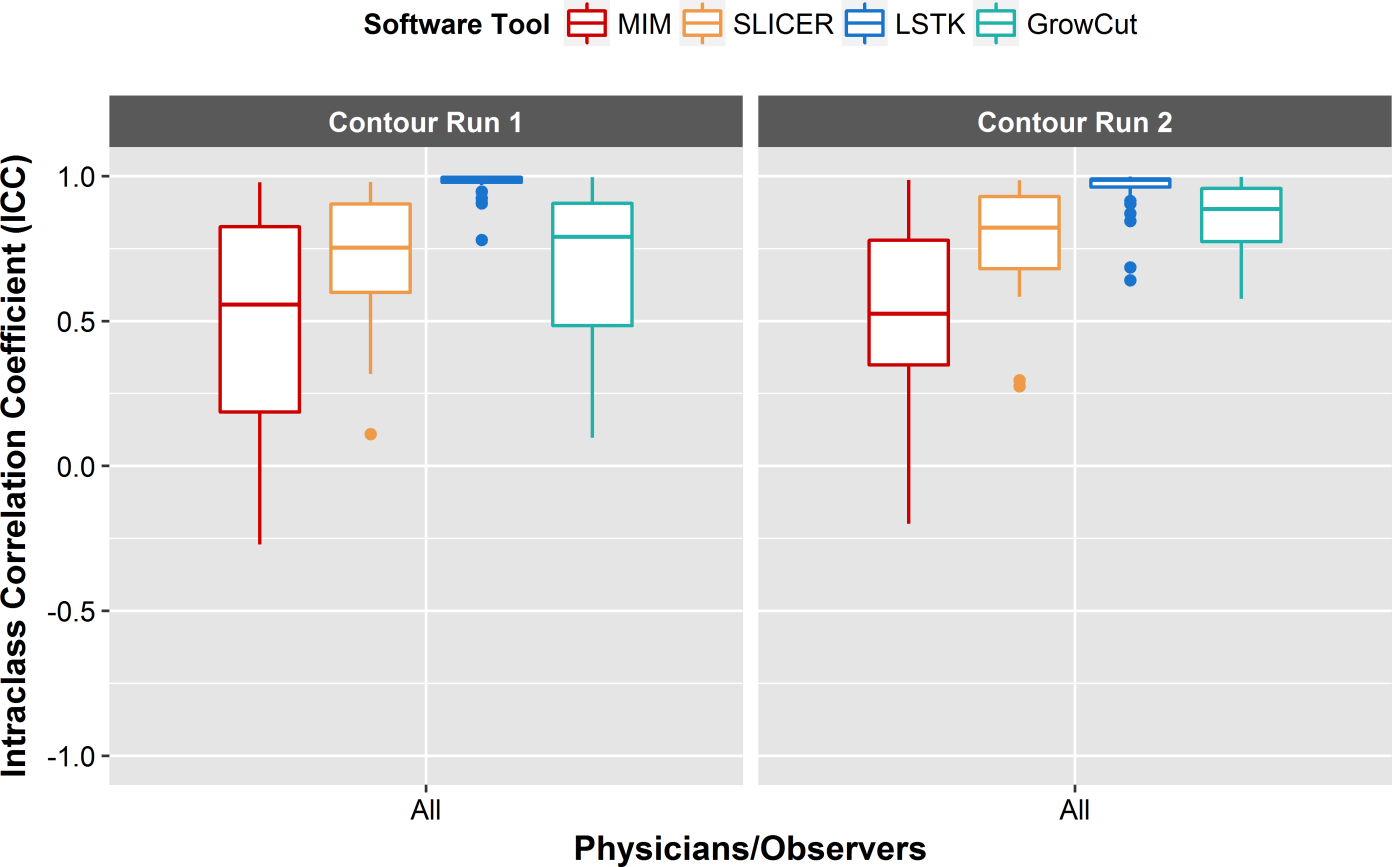
Inter-observer reliability. Box plot of ICCs for each inter-observer relationship. The ICC values were computed between all physician/observer contours for each feature. Each contour run and software tool combination is plotted along the *x*-axis. Inter-observer reliability was superior with LSTK compared with all other software tools.

For inter-observer variability, we wanted to evaluate whether features could be extracted reliably from tumor contours generated by multiple observers using a single software tool. For each feature, ICC values were calculated between the features generated by multiple users for each contour run and software tool combination. For both manual tools, the average ICC was less than 0.79 for both contour runs (Fig 9, Table 2). For the semi-automatic tools, GrowCut (0.70, 0.85) had inferior feature reliability compared with LSTK (0.98, 0.96). Moreover, LSTK had average ICC values that fell within the excellent ICC classification for contour run 1 and contour run 2. This shows that LSTK has superior feature reliability across observers compared with the other software tools used in this study.

#### Feature reproducibility: Inter-software

**Fig 10:**
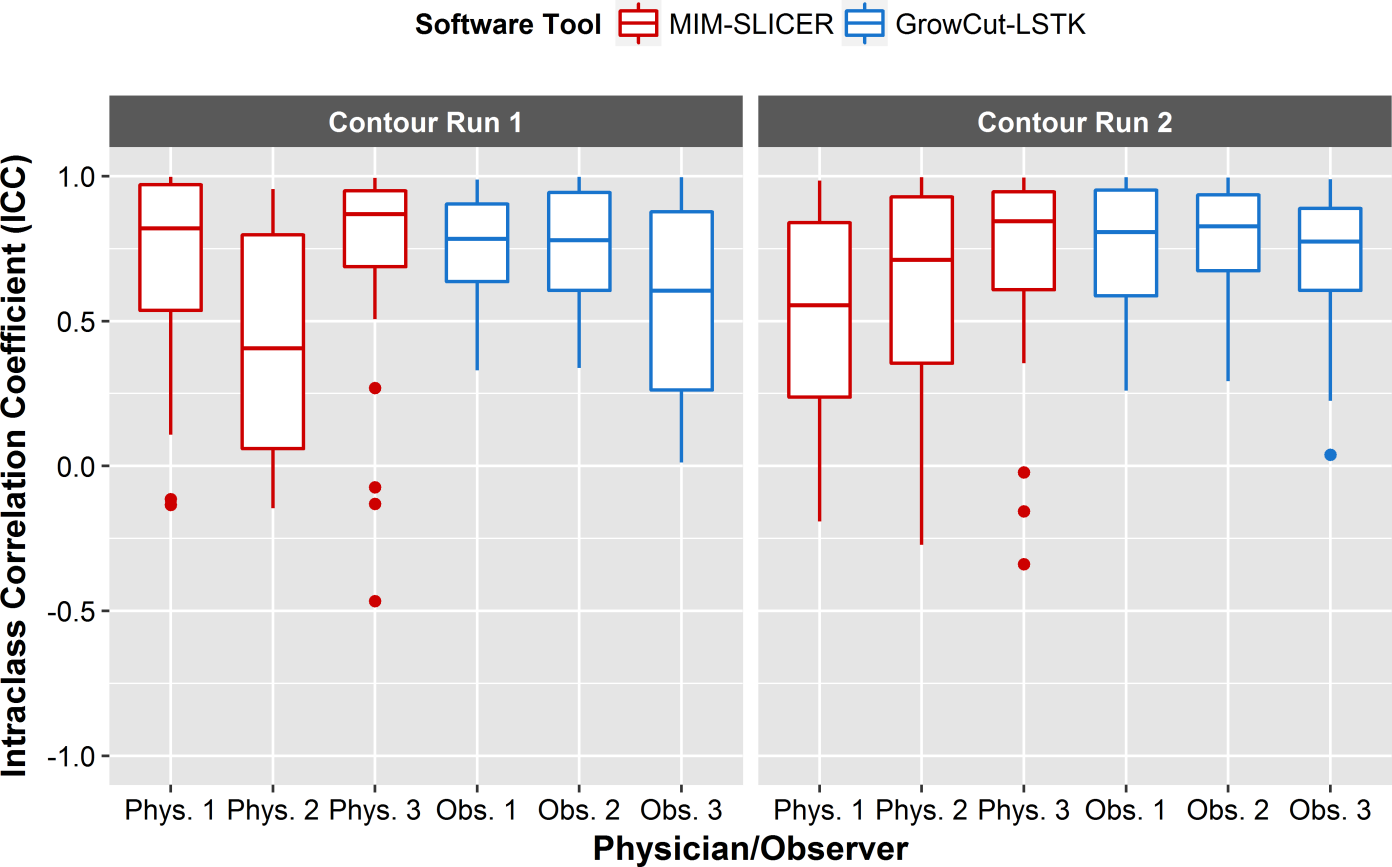
Inter-software reliability. Box plot of ICCs for each inter-software relationship. The ICC values were computed between contours generated by two different software tools for each feature. Each contour run and segmentation method combination is plotted along the *x*-axis. Inter-observer reliability was relatively low for all inter-software relationships, with the ICC values for many features falling within the poor classification.

For inter-software reliability, we sought to evaluate whether features could be extracted reliably from tumor contours generated by a single observer using multiple software tools. For each feature, ICC values were calculated between the features generated by multiple software tools for each user. For both manual and software methods, the average ICC was less than 0.78 for all physicians and observers (Fig 10, Table 2). Although 0.78 falls within the good reproducibility bounds, it is important to note that the confidence intervals for these results are very large (which could be attributable to the small sample size used in this study) and that for many features the lower bound of the confidence interval overlaps with the bounds of the ICC classification for poor reproducibility. These results indicated that different software tools do not yield reproducible features and should not be used interchangeably. This has also been concluded by other studies looking specifically at lung nodule volumes [36,37].

**Fig 11:**
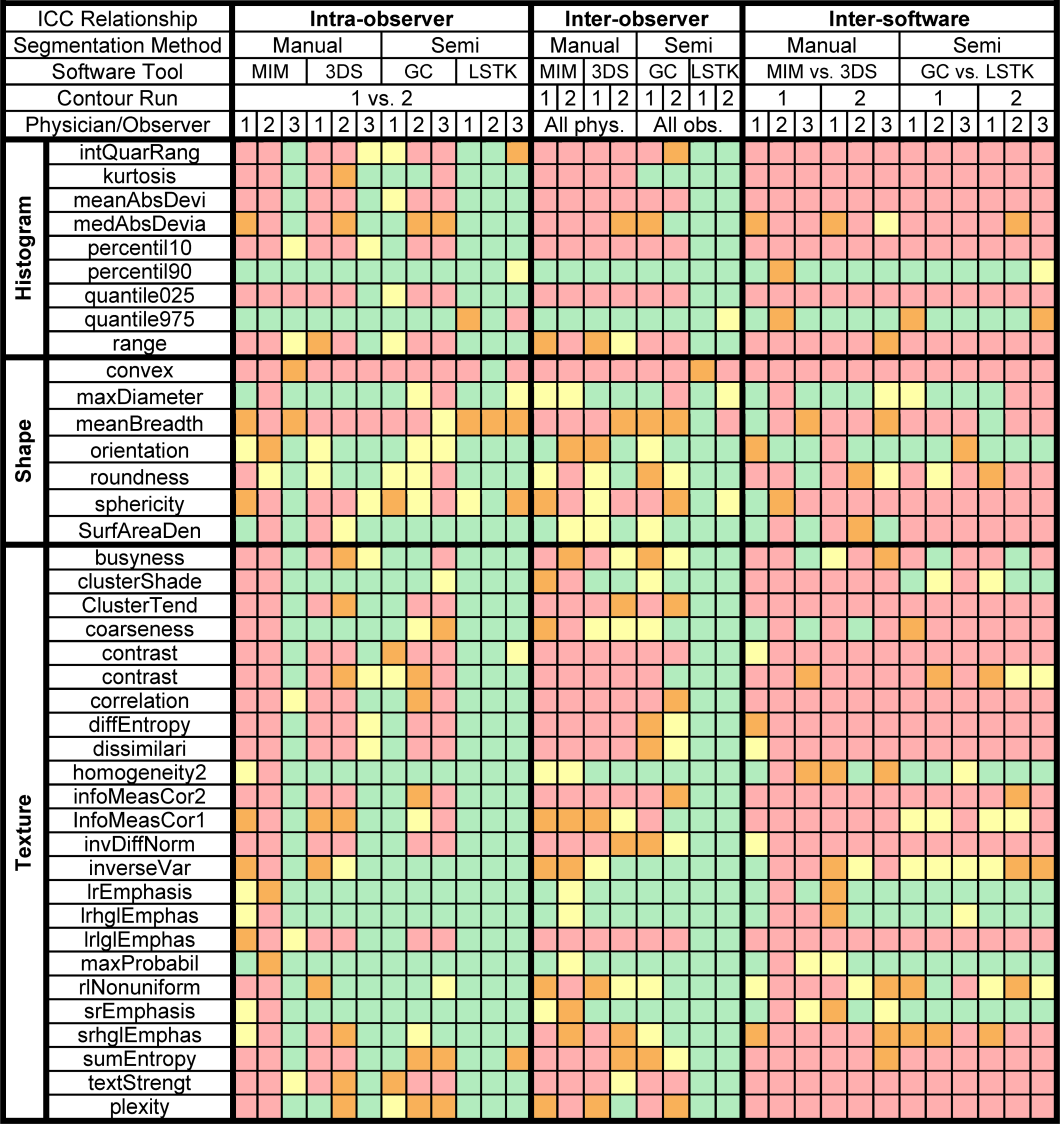
ICC classification of each radiomics feature for each ICC relationship. Red, orange, yellow, and green cells denote the ICC classifications of poor (*ICC* < 0.4), fair (0.4 ≤ *ICC* < 0.60), good (0.60 ≤ *ICC* < 0.75), and excellent (0.75 ≤ *ICC*) reproducibility, respectively [33].

Because the boxplots (Figs 8–10) show only the spread of ICC values for each ICC relationship, Fig 11 allows one to see the ICC classification of each feature for each ICC relationship. ICC values were sorted into their respective ICC classifications based on the lower bound of the 95% confidence interval of the ICC value (Fig 11). Koo et al recommends using the 95% confidence interval to evaluate the level of reliability rather than using the ICC estimate, as the ICC estimate is merely an expected value of the true ICC [38]. Once more, the results in Fig 11 further support the fact that LSTK has superior feature reproducibility, with 31 of the 40 features having lower bound values that fell within the excellent classification for all intra-observer and inter-observer relationships. These results showed that LSTK helps to improve feature reliability for many features across observers and for repeat measures performed by a single observer. Additionally, it can easily be noted that most features, irrespective of the segmentation method, contour run, or physician/observer, fell within the poor classification for feature reproducibility for all inter-software relationships.

#### Identifying reliable feature categories

**Fig 12:**
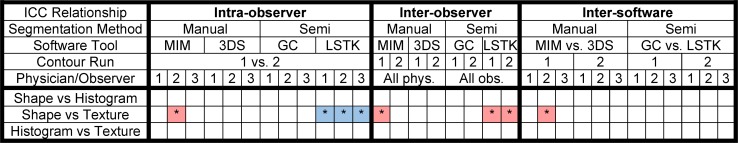
Wilcoxon rank sum results between intraclass correlation coefficients for different feature categories. Asterisks indicate that the median ICC was significantly different (p<0.05) between the two feature categories being compared. Blue cells indicate that the reproducibility of texture features was significantly less than the reproducibility of shape features. Red cells indicate that the reproducibility of texture features was significantly greater than the reproducibility of shape features.

In this part of the analysis, we wanted to evaluate whether a specific feature category was more reproducible than another feature category. The results for the Wilcoxon rank sum tests showed that for all ICC relationships, the reproducibility of shape features did not significantly differ from the reproducibility of histogram features, and that the reproducibility of histogram features did not significantly differ from the reproducibility of texture features (Fig 12). For assessing whether the reproducibility of shape features was significantly different from the reproducibility of texture features, only four ICC relationships had shape features that were significantly more reproducible than texture features, whereas three ICC relationships had shape features that were significantly less reproducible than texture features. Overall, no feature category was found to be more reproducible than another.

### Feature range analysis

**Fig 13:**
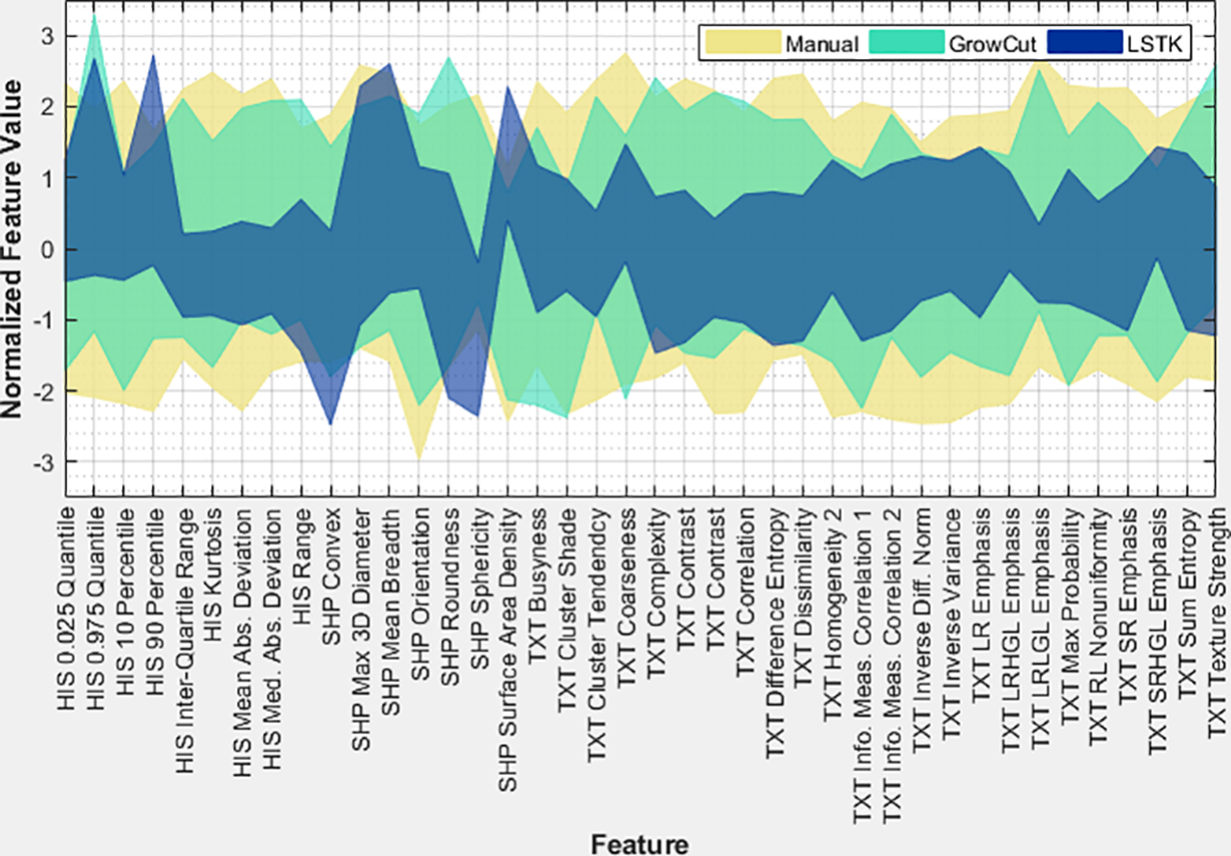
Normalized feature range. Comparison of normalized feature range between manual and semi-automatic methods using z-score normalization. The minimum and maximum values are plotted for each feature and segmentation method.

To assess the feature range for each feature, we plotted the minimum and maximum normalized feature values for each segmentation method (Fig 13). The semi-automatic contours had smaller feature ranges than the manual delineations for most of the features (except 7 features of a total of 40). Furthermore, when we compared the feature ranges for LSTK, all features had smaller feature ranges across observers than the manual delineations. Additionally, all but four features had ranges that overlapped with the manual ranges.

## Discussion

Tumor delineation is an important aspect of the radiomics workflow. Variation in contouring can affect the extracted feature values, which would undoubtedly influence subsequent steps in the radiomics workflow. Identifying contouring software tools that improve feature reliability helps to mitigate feature uncertainties that arise from inconsistent contouring. In this study, we evaluated the uncertainty of radiomics features from both manual and semi-automatic segmentation due to intra-observer, inter-observer, and inter-software reliability. We found that, using semi-automatic segmentation such as LSTK, observers without formal clinical training can generate contours that are comparable to manually drawn contours generated by formally trained physicians (Fig 4).

In terms of intra-observer reliability, we found that features extracted from LSTK contours were more reliable than those extracted from contours generated with other software tools for all observers (Fig 8, Table 2). In both semi-automatic segmentation tools, LSTK showed better intra-observer reliability than GrowCut because less human interaction was needed to generate contours with LSTK, which was exemplified by the improvement in intra-observer reliability from observer 3 (Table 2). For inter-observer reliability, we found that features extracted from LSTK contours were more reliable across observers than features extracted with all other software tools (Fig 9). Regarding inter-software reliability, we found that different software tools do not yield reproducible features, even when the same observer uses the two tools (Fig 10). In other words, segmentation tools cannot be used interchangeably if the contours will be used in subsequent radiomics studies. In addition, we also found that the feature range was smaller across observers for all features generated from LSTK contours than other contours (Fig 13), implying less uncertainty when the contours were generated with less human interaction. In other words, to minimize the uncertainty in radiomics studies, one should adhere to a single contouring approach and automate the contouring process as much as possible. Additionally, for the most part, we found that no feature category was found to be more reproducible than another (Fig 12).

Our findings agree with a previously conducted study which found that features were less reliable when extracted from segmentations generated with different algorithms (similar to our inter-software relationship) compared with features extracted from segmentations from repeat runs of the same algorithm (similar to our intra-observer relationship) [17]. The difference between our study and the study by Kalpathy-Cramer et al is that we also looked at the effect of different observers using the same segmentation tool. This is an important interaction to assess because different observers, depending on their training and familiarity with the segmentation tool, may use the same tool differently which can affect the final segmentation.

There are three main limitations of this study. The first limitation is that a small patient population was used. Sample size is an important factor to consider when using inferential statistics such as the ICC. Small sample sizes lack power and can result in large confidence intervals [39]. The negative ICC values observed in this study could be caused by the insufficient sample size as well. Future studies with larger sample sizes may help to reduce wide confidence intervals. Despite the small sample size, however, the width of the confidence intervals was narrower for all features extracted from LSTK contours compared with the other software tools for all intra-observer and inter-observer relationships.

The second limitation is that the ICC (as is the case for any reliability measure) depends on the heterogeneity of the tumors of the patient population in the study [40,41]. Populations that are more heterogeneous (where the between-subject standard deviation is larger) will yield higher ICC values than more homogeneous populations. Because of these limitations, we reported confidence intervals of the ICC averages (Table 2), as well as the tumor volume range (1.15 cm^3^ to 10.53 cm^3^) for this patient population to give an idea of the between-patient tumor heterogeneity.

The third limitation is that we tested only the most popular radiomics features instead of an exhaustive list of radiomics features. One group of radiomics features that is worth mentioning is the edge sharpness features [42]. On the basis of its construction, we expect edge features to be highly correlated with shape features under test. For example, the shape features sphericity and compactness would be influenced by the smoothness of the tumor’s boundary, with smoother boundaries yielding larger feature values and rougher boundaries yielding smaller feature values. Because both shape and edge features are calculated from the tumor boundary, we believe that edge features may exhibit similar feature variability due to segmentation differences as we observed with shape features.

Although we showed that LSTK improves feature reliability (within and across observers), its effect on outcome modeling has not been evaluated. Radiomics features alone are not very meaningful. After feature extraction, features are often evaluated to see if they correlate with prognostic or predictive factors. An important future study would be to evaluate the effect that contouring can play in building outcome models. It has been shown from this study and other studies that semi-automatic tools improve feature reliability [13–16]; however, to the best of our knowledge the effects of these tools on building outcome models have yet to be studied. Also, semi-automatic tools that yield accurate segmentations and improve segmentation consistency within and across observers are not only helpful for feature reliability studies but also can help with subsequent studies that utilize tumor contours in their analysis. Examples of such studies include but are not limited to longitudinal radiomics studies (delta-radiomics) and longitudinal clinical studies [7,43] that assess tumor response where contours may be generated across different observers or at different time points by a given observer.

## Conclusion

Our findings showed that radiomics features computed from semi-automatic segmented volumes have better feature reproducibility and reliability than those computed from manual segmented volumes. In semi-automatic segmentation, the tool with less human interaction (i.e. LSTK) resulted in better feature reliability as well. Our results also showed that with semi-automatic segmentation tools, observers without formal clinical training were comparable to physicians in evaluating tumor segmentation. Our findings suggest the need of developing fully automatic segmentation tools (without any user input) for radiomics studies in order to minimize the impact from contouring uncertainty and to improve feature reproducibility and repeatability for subsequent analysis such as radiomics outcome studies or longitudinal clinical studies that assess tumor response.
